# Identification of Hypertension Predictors and Application to Hypertension Prediction in an Urban Han Chinese Population: A Longitudinal Study, 2005–2010

**DOI:** 10.5888/pcd12.150192

**Published:** 2015-10-29

**Authors:** Wenchao Zhang, Linping Wang, Yafei Chen, Fang Tang, Fuzhong Xue, Chengqi Zhang

**Affiliations:** Author Affiliations: Wenchao Zhang, Yafei Chen, Fuzhong Xue, Department of Biostatistics, School of Public Health, Shandong University, Jinan, China; Linping Wang, Fang Tang, Health Management Center, Shandong Provincial QianFoShan Hospital, Jinan, China.

## Abstract

**Introduction:**

Research suggests that targeting high-risk, nonhypertensive patients for preventive intervention may delay the onset of hypertension. We aimed to develop a biomarker-based risk prediction model for assessing hypertension risk in an urban Han Chinese population.

**Methods:**

We analyzed data from 26,496 people with hypertension to extract factors from 11 check-up biomarkers. Then, depending on a 5-year follow-up cohort, a Cox model for predicting hypertension development was built by using extracted factors as predictors. Finally, we created a hypertension synthetic predictor (HSP) by weighting each factor with its risk for hypertension to develop a risk assessment matrix.

**Results:**

After factor analysis, 5 risk factors were extracted from data for both men and women. After a 5-year follow-up, the cohort of participants had an area under receiver operating characteristic curve (area under the curve [AUC]) with an odds ratio (OR) of 0.755 (95% confidence interval [CI], 0.746–0.763) for men and an OR of 0.801 (95% CI, 0.792–0.810) for women. After tenfold cross validation, the AUC was still high, with 0.755 (95% CI, 0.746–0.763) for men and 0.800 (95% CI, 0.791–0.810) for women. An HSP-based 5-year risk matrix provided a convenient tool for risk appraisal.

**Conclusion:**

Hypertension could be explained by 5 factors in a population sample of Chinese urban Han. The HSP may be useful in predicting hypertension.

## Introduction

Hypertension is a worldwide public health challenge because of its high frequency and concomitant risks of cardiovascular disease and renal disease (1). Many studies have demonstrated that lifestyle modification can prevent high blood pressure, providing a rationale for the identification of high-risk participants so that early lifestyle intervention strategies can be implemented to prevent hypertension (2–4). In recent years, researchers have established hypertension prediction models for different populations, including Americans (5–8), Iranians (9), and Chinese (10,11). The 2 studies of a Chinese population prediction model (10,11) had areas under the curve (AUCs) that ranged from 71.6% to 73.5%. Inability to incorporate enough laboratory biomarkers and existing recall bias from questionnaire variables limited the effect of the 2 prediction models. This study corrects these limitations. Prevalence, awareness, treatment, and control of hypertension in China, a developing country, differs from that of developed countries (12,13). The purpose of this study was to develop a biomarker-based risk-prediction model for hypertension in a population of urban Han Chinese adults.

## Methods

Of 95,785 people aged 18 to 88 years who received annual medical examinations from 2005 through 2010 at the Center for Health Management of Shandong Provincial QianFoShan Hospital and Shandong Provincial Hospital, 26,496 were diagnosed with hypertension at their first check-up. Of 69,289 people without hypertension at baseline, 17,471 (10,239 men and 7,232 women) who received annual clinical and laboratory examinations were selected as a 5-year follow-up cohort.

We selected 11 biomarkers to analyze from the routine health check-ups. They were body mass index (BMI), systolic blood pressure (SBP), diastolic blood pressure (DBP), fasting blood glucose (FBG), triglycerides (TG), high-density lipoprotein cholesterol (HDL-C), hemoglobin (Hb), hematocrit (HCT), white blood cell count (WBC), lymphocyte count (LC), and neutrophil granulocyte count (NGC). These biomarkers have been previously associated with hypertension: BMI (14–16), SBP and DBP (17), FBG (18), TG (16), HDL-C (19,20), Hb and HCT (21), and WBC, LC, and NGC (22).

We measured the height and weight of participants who were wearing light clothing and no shoes. BMI was calculated as weight (kg) divided by height squared (m^2^). SBP and DBP were measured using Omron HEM-907 (QuickMedical) by the cuff-oscillometric method in the right arm of seated participants after a 5-minute rest period. Two measurements were taken, and the 2 BP values were averaged. Peripheral blood samples were obtained in the morning after a 12-hour fast to measure the following biomarkers: FBG, TG, HDL-C, Hb, HCT, WBC, LC, and NGC. The study was approved by the Ethics Committee of School of Public Health, Shandong University, and written informed consent was obtained from all participants.

Hypertension was defined as diastolic blood pressure of 90 mm Hg or more, systolic blood pressure of 140 mm Hg or more, or reported use of medication known to treat hypertension. Age and biomarkers were presented by mean (standard deviation), and Student’s *t* test was used to distinguish between participants with and without baseline hypertension.

First, to eliminate multicollinearity between the routine check-up biomarkers and to extract risk-related factors of hypertension from them, we used factor analysis with principal component algorithm and varimax rotation from correlation matrix. The criteria for retaining factors were set up as eigenvalue of higher than 1. Further analytical interpretation used biomarkers that share a factor loading of at least 0.50. Second, on the basis of the cohort design, the Cox proportional hazards regression model was built between the hazard function of hypertension and the extracted latent factors:

h_i_(t) = h_0_(t)exp(β_0_age_i_+β_1_F_i1_+β_2_F_i2_+…+β_k_F_ik_)

where h_i_(t) is the hazard rate for the i^th^ subject at time t, and h_0_(t) is the baseline hazard at time t. Then, a hypertension synthetic predictor (*HSP*) was derived by weighting each factor with its regression coefficients using the formula

*HSP* = β_1_F_1_+β_2_F_2_+…+β_k_F_k_.

After that, we calculated an *HSP* for each participant. Third, on the basis of the cohort design, the Cox proportional hazards regression model was built again between the hazard function of hypertension and the calculated *HSP*:

h_i_(t) = h_0_(t)exp(*θ*_0_ *age* + *θ*_1_ *HSP*)

The predictive probability of hypertension at year *t* was calculated by the following formula:


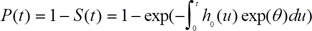


where *θ* = *θ*_0_ *age* + *θ*_1_ *HSP*

Fourth, we used MedCalc software (23) for analysis of receiver operator characteristics (ROC) curve, sensitivity, specificity, and significance (*P* value) to evaluate the prediction effect. Finally, for each participant from the 5-year cohort study, we calculated relative absolute risk (RAR) using the following equation:


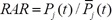


where *P_j_* (*t*) known as absolute risk (AR), was the probability of hypertension at year *t*, in which *j* denoted the participant’s age. 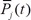 signified the average probability of hypertension at year *t* in *j^th^* age, which can be calculated by the following model:


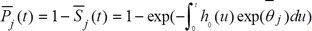


where 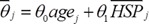, 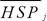 was the mean of *HSP* in *j^th^* age.

All data analyses in this study were conducted for both men and women. We used ArcGIS 9.1 (Esri) to depict the HSP-based 5-year risk matrix for hypertension risk appraisal. All statistical analyses were performed using SAS version 9.2 (SAS Institute, Inc), and significance was set at *P* < .05.

## Results

**Figure 1:**
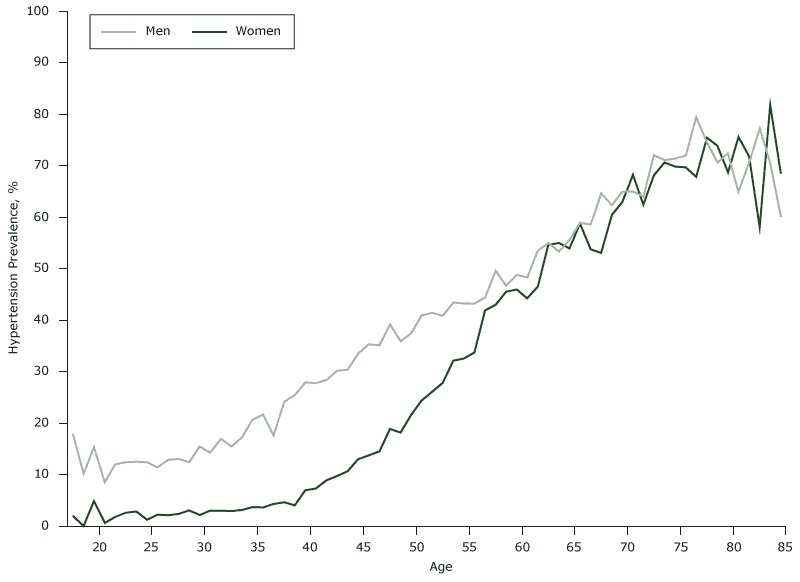
Prevalence of hypertension, by age, at baseline, in an urban Han population, China, 2005–2010. **Table:** 

In our study, 26,496 of 95,785 participants had hypertension at baseline, a prevalence of 27.7% (32.6% for men and 19.5% for women). Although hypertension prevalence increased with age in both men and women (Figure 1), it was higher in men than women before age 60 and was similar after age 60. Of the 3,793 participants (2,894 men and 899 women) who did not have hypertension at baseline but had hypertension at the end of year 5, the cumulative incidence was 21.7% (3,793 of 17,471). We calculated the distribution of age and 11 biomarkers among participants with and without baseline hypertension (Table 1), and all variables differed significantly for participants with and without baseline hypertension. Results of the analysis were used to create a correlation matrix for the 11 biomarkers (Appendix A).

Age, y	Hypertension Prevalence, %	
	Men	Women
18	17.91	2.08
19	10.23	0
20	15.44	4.92
21	8.6	0.69
22	12.02	1.78
23	12.46	2.65
24	12.54	2.91
25	12.46	1.27
26	11.47	2.29
27	12.91	2.2
28	13.09	2.44
29	12.45	3.09
30	15.51	2.2
31	14.29	3.05
32	16.94	3.04
33	15.5	2.93
34	17.29	3.17
35	20.71	3.74
36	21.73	3.7
37	17.62	4.38
38	24.19	4.66
39	25.43	4.06
40	27.93	6.99
41	27.77	7.32
42	28.43	8.98
43	30.2	9.79
44	30.46	10.73
45	33.55	13.02
46	35.34	13.72
47	35.13	14.55
48	39.21	18.88
49	35.91	18.22
50	37.45	21.62
51	40.97	24.39
52	41.46	26.11
53	40.85	27.83
54	43.43	32.16
55	43.27	32.58
56	43.24	33.78
57	44.41	41.92
58	49.64	42.96
59	46.72	45.53
60	48.8	45.95
61	48.34	44.26
62	53.5	46.58
63	55	54.62
64	53.31	54.97
65	55.68	53.95
66	58.91	58.79
67	58.59	53.8
68	64.62	53.09
69	62.35	60.38
70	64.94	62.96
71	64.99	68.25
72	63.95	62.41
73	71.99	68.16
74	71.12	70.61
75	71.35	69.83
76	71.97	69.68
77	79.4	67.8
78	74.42	75.42
79	70.62	73.86
80	72.4	68.63
81	64.89	75.61
82	70.68	71.74
83	77.27	58.06
84	70.59	81.82
85	60	68.42

**Table 1:** Distribution of Age and the 11 Biomarkers for Participants With and Without Baseline Hypertension, China, 2005–2010

Variable	Without Baseline Hypertension (n = 69,236)	With Baseline Hypertension (n = 26,478)	*P* Values^a^
	Mean (SD)		
Age, y	41.37 (12.37)	53.62 (14.24)	<.001
Body mass index, kg/m^2^	24.27 (3.59)	26.86 (3.49)	<.001
Systolic blood pressure, mmHg	117.4 (12.53)	149.2 (16.29)	<.001
Diastolic blood pressure, mmHg	70.84 (9.24)	86.30 (12.73)	<.001
Fasting blood-glucose, mmol/L	5.20 (1.11)	5.80 (1.60)	<.001
Triglycerides, mmol/L	1.49 (1.32)	2.06 (1.79)	<.001
High-density lipoprotein cholesterol, mmol/L	1.33 (0.33)	1.30 (0.36)	<.001
Hemoglobin, g/L	146.7 (15.51)	151.1 (14.40)	<.001
Hematocrit, %	43.61 (4.05)	44.74 (3.79)	<.001
White blood cell count, 10^9^/L	6.46 (1.59)	6.85 (1.68)	<.001
Lymphocyte, 10^9^/L	2.07 (0.61)	2.15 (0.71)	<.001
Neutrophil granulocyte, 10^9^/L	3.84 (1.23)	4.11 (1.26)	<.001

a *P* values were calculated using Student’s *t* test.

 After exploring factor analysis (EFA), 5 latent factors were extracted from 11 biomarkers. Combined with explained variance and cumulative variance, factor loadings by principal component analysis with varimax rotation (Table 2). Five latent risk-related factors could explain 72.21% of total variance for men and 72.47% for women: inflammatory factor (IF), blood viscidity factor (BVF), insulin resistance factor (IRF), blood pressure factor (BPF), and lipid resistance factor (LRF). Of the 5 factors, IF was contributed by WBC and LC and NGC, BVF by Hb and HCT, IRF by FBG and TG, BPF by SBP and DBP, and LRF by BMI and HDL-C.

**Table 2:** Factor Loadings by Principal Component Analysis With Varimax Rotation on 11 Routine Health Check-up Biomarkers in Hypertension Patients, China, 2005–2010

Biomarker	Factor									
	Men					Women				
	Inflam-matory	Blood Viscidity	IR	BP	Fat Resistance	Inflam-matory	Blood Viscidity	BP	IR	Fat Resistance
BMI	0.0630	0.0978	0.3681	0.1534	−0.6950^a^	0.1000	0.0486	0.3131	0.2556	−0.6194^a^
SBP	0.0527	−0.1677	−0.0245	0.8540^a^	0.0113	0.0419	−0.0574	0.7988^a^	0.1902	0.0234
DBP	−0.0003	0.3203	0.1370	0.7064^a^	−0.0595	0.0114	0.1837	0.7549^a^	−0.1657	−0.1249
FBG	0.0342	−0.0994	0.6782^a^	0.0696	−0.0777	0.0785	0.0423	0.0011	0.7670^a^	−0.0739
TG	0.0836	0.1380	0.7850^a^	−0.0029	0.1003	0.0807	0.0798	0.0356	0.7250^a^	−0.0163
HDL-C	−0.0650	−0.0171	0.2468	0.0761	0.8323^a^	−0.0431	−0.0120	0.0782	0.0574	0.8778^a^
Hb	0.0627	0.9574^a^	0.0450	0.0333	−0.0550	0.0639	0.9705^a^	0.0542	0.0920	−0.0403
HCT	0.1242	0.9472^a^	−0.0234	0.0332	−0.0425	0.1146	0.9651^a^	0.0674	0.0640	−0.0138
WBC	0.9906^a^	0.0718	0.0431	0.0355	−0.0512	0.9872^a^	0.0747	0.0170	0.0626	−0.0957
LC	0.6468^a^	0.0577	0.0850	−0.0199	−0.0306	0.6524^a^	0.0637	0.0837	0.1971	0.0990
NGC	0.8548^a^	0.0544	0.0036	0.0589	−0.0454	0.8577^a^	0.0571	−0.0277	−0.0327	−0.1728
% Variance explained	0.2354	0.1578	0.1260	0.1060	0.0969	0.2464	0.1558	0.1237	0.1037	0.0951
Cumulative variance	0.2354	0.3932	0.5192	0.6252	0.7221	0.2464	0.4022	0.5259	0.6296	0.7247

Abbreviations: BMI, body mass index; BP, blood pressure; DBP, diastolic blood pressure; FBG, fasting blood glucose; Hb, hemoglobin; HCT, hematocrit; HDL-C, high-density lipoprotein cholesterol; IR, insulin resistance; LC, lymphocyte; NGC, neutrophil granulocyte; SBP, systolic blood pressure; TG, triglycerides; WBC, white blood cell count.

a Factor loading >0.5.

**Figure 2:**
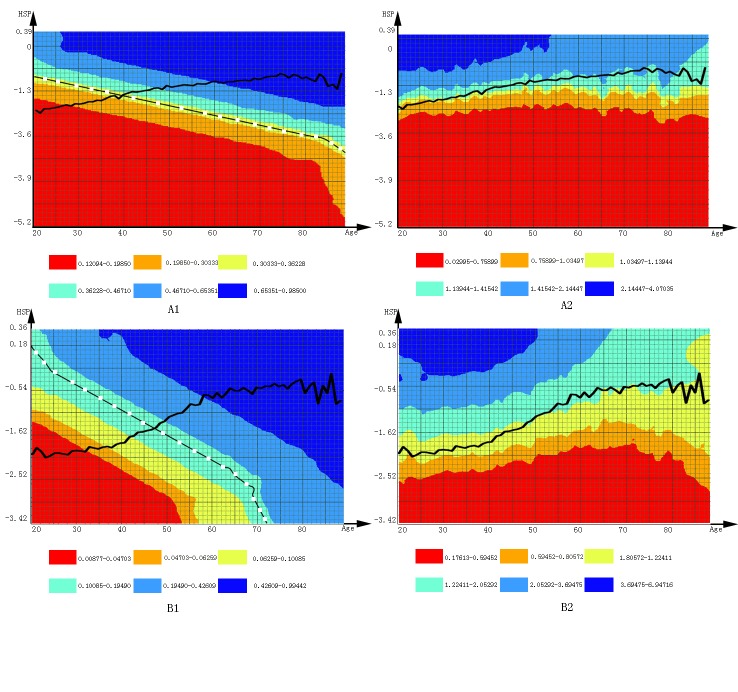
The 5-year risk matrix for risk appraisal of hypertension by sex. Graphs A1 and B1 are absolute risk matrices for men and women, respectively; graphs A2 and B2 are relative absolute risk matrices for men and women, respectively. The dashed lines indicate discrimination criteria of absolute risk for predicting hypertension; the curved lines indicate mean absolute risk in the population. Abbreviation: HSP, hypertension synthetic predictor.

ROC curves for hypertension prediction models are in Figure S1 (Appendix B). The AUC was up to 75.5% for men and 80.1% for women (Figure 2, graphs A1 and A2 for men and graphs B1 and B2 for women), and was 75.5% and 80.0% after tenfold cross validation. These matrices provide a convenient tool for hypertension prediction in clinical and health management. For example, if a man aged 40 came to a hospital for a checkup, and 11 routine health check-up biomarkers (BMI, SBP, DBP, FBG, TG, HDL-C, Hb, HCT, WBC, LC, NGC) were tested, his HSP could be calculated using the formula in Figure 2. After that, we find his absolute risk (AR) and RAR in (graphs A1 and A2) through his age and his HSP. AR shows his predictive probabilities for hypertension are more than 5, and RAR shows his hypertension risk compared with his peers (people aged 40).

## Discussion

In our study, the prevalence of hypertension was higher among men (32.6%) than women (19.5%) at baseline. However, hypertension prevalence changes with age. Hypertension prevalence rises more steeply in aging women than in men, perhaps because of hormonal changes during menopause (24–26).

Distribution of age and 11 routine health check-up biomarkers of participants with and without baseline hypertension differed significantly. After factor analysis, 5 latent factors were extracted from 11 biomarkers, not only eliminating the multicollinearity, but also explaining the specific pathogenesis of hypertension. The 5 factors were used to predict hypertension in the following prediction model. The 5 factors are the inflammatory factor (IF), blood viscidity factor (BVF), insulin resistance factor (IRF), blood pressure factor (BPF), and lipid resistance factor (LRF) in both men and women, according to our analysis. The cumulative explained variances of the 5 latent factors were 72.21% for men and 72.47% for women. IF and BVF in particular were identified as the key factors for the variation of hypertension (IF contributes 23.54% for men and 24.64% for women; BVF, 15.78% for men and 15.58% for women). Similar results have been found in other studies. Evidence from human and animal studies suggests that inflammation leads to the development of hypertension and that oxidative stress and endothelial dysfunction are involved in the inflammatory cascade (27). The elevation in blood viscosity could increase peripheral resistance and play a role in the pathogenesis of essential hypertension (28–30).

Two hypertension prediction models have been developed in Chinese populations. Although the power of these prediction models (AUC range: 71.6%–73.5%) was acceptable, their risk algorithm and visualization of risk assessment still had room for improvement. Our HSP-based prediction model had better prediction effect (ROC, 75.5% for men and 80.1% for women; ROC after tenfold cross validation, 75.5% for men and 80.0% for women.). One reason our results were more accurate is because we used laboratory biomarkers rather than questionnaire variables to avoid recall bias. Another reason is that we used both factor analysis and the Cox model, producing a better model for hypertension prediction. Finally, we developed a risk matrix with AR and RAR to represent risk assessment, which was convenient for practical application (Figure 2). For example, men and women who receive a routine health check-up can learn their AR from the risk matrix. Comparing their results with those of other same-age participants may warn patients of their risk and guide their choice of nonpharmacologic measures to prevent hypertension. A limitation to our study was that all participants were urban Han Chinese adults; therefore, our results may not be generalizable to other populations. More validation studies for these prediction models are needed.

This is the first hypertension prediction model developed for urban Han Chinese adults. Because of the large sample size, the estimates from our prediction model were stable, as demonstrated by the tenfold cross validation. Physicians can use the HSP-based 5-year hypertension risk matrix to measure patients’ risk for hypertension, inform patients of their risks, help them choose appropriate nonpharmacologic measures to prevent hypertension, and aid in clinical counselling and decision making.

## Appendices

Appendix A. Correlation Matrix Between 11 Biomarkers, Urban Han Population, China, 2005–2010. This file is available for download as a Microsoft Word document.

Appendix B. Receiver Operator Characteristics (ROC) Curves for Hypertension Prediction Models, Urban Han Population, China, 2005–2010.
