# Supplementary material for: Identification of Hypertension Predictors and Application to Hypertension Prediction in an Urban Han Chinese Population: A Longitudinal Study, 2005–2010
Source: Prev Chronic Dis. 2015 Oct 29;12:E184. doi: 10.5888/pcd12.150192 (PMC4663898; doi:10.5888/pcd12.150192)
Supplement: Supplementary file 1 [file 15_0192_AppendixA.docx]

# Table S1 Correlation matrix between eleven biomarkers.

|  | **BMI** | **SBP** | **DBP** | **FBG** | **TG** | **HDL_C** | **Hb** | **HCT** | **WBC** | **LC** | **NGC** |
| --- | --- | --- | --- | --- | --- | --- | --- | --- | --- | --- | --- |
| **BMI** | 1 | 0.410774 | 0.400376 | 0.214113 | 0.277415 | -0.2909 | 0.276545 | 0.274611 | 0.200082 | 0.137655 | 0.161724 |
| **SBP** |  | 1 | 0.678504 | 0.257851 | 0.206577 | -0.07871 | 0.205013 | 0.210036 | 0.141939 | 0.093565 | 0.117625 |
| **DBP** |  |  | 1 | 0.190621 | 0.25983 | -0.10132 | 0.328413 | 0.316136 | 0.154126 | 0.094722 | 0.131809 |
| **FBG** |  |  |  | 1 | 0.240532 | -0.02941 | 0.106177 | 0.083612 | 0.095851 | 0.086297 | 0.068949 |
| **TG** |  |  |  |  | 1 | -0.03286 | 0.239454 | 0.212709 | 0.184042 | 0.139523 | 0.144352 |
| **HDL-C** |  |  |  |  |  | 1 | -0.24418 | -0.22092 | -0.15294 | -0.07862 | -0.1384 |
| **Hb** |  |  |  |  |  |  | 1 | 0.946648 | 0.232583 | 0.146947 | 0.189309 |
| **HCT** |  |  |  |  |  |  |  | 1 | 0.264819 | 0.196211 | 0.197778 |
| **WBC** |  |  |  |  |  |  |  |  | 1 | 0.592657 | 0.895534 |
| **LC** |  |  |  |  |  |  |  |  |  | 1 | 0.192115 |
| **NGC** |  |  |  |  |  |  |  |  |  |  | 1 |

All *p* values were P≤0.05.
